# LinkImpute: Fast and Accurate Genotype Imputation for Nonmodel Organisms

**DOI:** 10.1534/g3.115.021667

**Published:** 2015-09-15

**Authors:** Daniel Money, Kyle Gardner, Zoë Migicovsky, Heidi Schwaninger, Gan-Yuan Zhong, Sean Myles

**Affiliations:** * Department of Plant and Animal Sciences, Faculty of Agriculture, Dalhousie University, Truro, Nova Scotia, B2N 5E3, Canada; † USDA-ARS Plant Genetic Resources Unit, Geneva, New York 14456

**Keywords:** imputation, SNP, genotyping by sequencing, apple

## Abstract

Obtaining genome-wide genotype data from a set of individuals is the first step in many genomic studies, including genome-wide association and genomic selection. All genotyping methods suffer from some level of missing data, and genotype imputation can be used to fill in the missing data and improve the power of downstream analyses. Model organisms like human and cattle benefit from high-quality reference genomes and panels of reference genotypes that aid in imputation accuracy. In nonmodel organisms, however, genetic and physical maps often are either of poor quality or are completely absent, and there are no panels of reference genotypes available. There is therefore a need for imputation methods designed specifically for nonmodel organisms in which genomic resources are poorly developed and marker order is unreliable or unknown. Here we introduce LinkImpute, a software package based on a *k*-nearest neighbor genotype imputation method, LD-kNNi, which is designed for unordered markers. No physical or genetic maps are required, and it is designed to work on unphased genotype data from heterozygous species. It exploits the fact that markers useful for imputation often are not physically close to the missing genotype but rather distributed throughout the genome. Using genotyping-by-sequencing data from diverse and heterozygous accessions of apples, grapes, and maize, we compare LD-kNNi with several genotype imputation methods and show that LD-kNNi is fast, comparable in accuracy to the best-existing methods, and exhibits the least bias in allele frequency estimates.

A primary goal of genomic research is to establish associations between genotypes and phenotypes. Our understanding of human disease has been significantly accelerated through studies linking genotypes with disease phenotypes, primarily through the use of genome-wide association (GWA) (*e.g.*, Altshuler *et al.* 2008). Such associations also are essential for accelerating breeding in agricultural species. For example, genome-wide, single-nucleotide polymorphism (SNP) data are used routinely in cattle to accelerate improvement with marker-assisted selection and genomic selection (GS) (*e.g.*, Hayes *et al.* 2009). The discovery and exploitation of genotype−phenotype associations in an increasing number of agricultural species has the potential to dramatically accelerate food improvement (McClure *et al.* 2014).

No matter what the focal species is, the discovery of novel genotype−phenotype relationships most often requires genome-wide genotype data from a large number of samples. To date, genotyping microarrays have been the technology of choice for acquiring these data. Arrays are widely used for GWA studies in humans (Stranger *et al.* 2011) and have also been developed for several agricultural species [*e.g.*, cattle (Matukumalli *et al.* 2009), rice (Zhao *et al.* 2011), grape (Myles *et al.* 2010), and apple (Chagné *et al.* 2012)]. Although arrays have proven to be effective in a few species, next-generation DNA sequencing is becoming the method of choice for generating genome-wide genotype data in many organisms. Methods that use reduced representation libraries and multiplex barcoding have been developed to generate genome-wide genotype data from next-generation DNA sequencing that are similar to the data generated from arrays and are suitable for GWA and GS. For example, genotyping-by-sequencing (GBS) (Elshire *et al.* 2011) produces genotype data useful for GS in wheat (Poland *et al.* 2012) and drastically reduces the cost of linkage mapping in apple compared with arrays (Gardner *et al.* 2014).

Although genotyping microarrays produce some missing data, GBS produces an extremely sparse genotype matrix with mostly missing data: often hundreds of thousands of SNPs are discovered, but only a small fraction of the SNPs (*e.g.*, <10%) pass missing data thresholds (Gardner *et al.* 2014). Imputing the missing genotypes has been shown to improve the power of methods such as GWA (Li *et al.* 2009) and so genotype imputation is becoming an increasingly important component of studies into genotype−phenotype relationships, especially when data are collected using methods like GBS.

Most existing genotype imputation methods, such as Beagle (Browning and Browning 2007) and fastPHASE (Scheet and Stephens 2006), rely on SNPs ordered according to a genetic or physical map. These methods are most often used to impute genotypes in species with large amounts of high-quality genotype data, including reference genomes and reference genotype panels, like humans (*e.g.*, International HapMap 3 Consortium 2010) and cattle (*e.g.*, Bovine HapMap Consortium 2009). These methods first phase the genotype data and take the phased haplotype information into consideration when inferring missing genotypes. Generic imputation methods that do not rely on phasing, such as *k*-nearest neighbors (Troyanskaya *et al.* 2001) or Missing Forest (MF) (Stekhoven and Bühlmann 2012), are not specifically designed for genotype data but can be used to impute missing genotypes. Although there have been comparisons between generic imputation methods (Stekhoven and Bühlmann 2012; Rutkoski *et al.* 2013) and between genotype-specific methods (Browning and Browning 2007; *e.g.*, Marchini and Howie 2010), there has been little comparison between the two groups of algorithms to date.

Here we introduce LD-kNNi, an imputation algorithm based on the *k*-nearest neighbors imputation (kNNi) method (Troyanskaya *et al.* 2001), which takes into account the linkage disequilibrium (LD) between SNPs when choosing the nearest neighbors. Critically, our algorithm does not require ordered SNPs, unlike most existing genotype specific methods such as Beagle and fastPHASE. We compare the performance of our new method to several existing methods by using genome-wide SNP data from apple, maize, and grape.

## Materials and Methods

### LD-kNNi

kNNi is a commonly used imputation method that has been used previously for genotype imputation (Troyanskaya *et al.* 2001) and has recently been extended to categorical data (Schwender 2012). In this work we only consider biallelic SNPs and code the genotypes numerically as 0 (homozygous major allele), 1 (heterozygous), and 2 (homozygous minor allele).

To impute a genotype at SNP *p_j_* in sample *s_i_* the categorical kNNi algorithm (henceforth simply kNNi) first calculates a distance from the sample to every other sample. We use the taxicab distance, where the distance, *d_n_* (*s*_1_, *s*_2_) between any two samples *s*_1_ and *s*_2_ is given by the following equation:

$$d_{n}\left(s_{1},s_{2}\right)=\frac{1}{n}\underset{p\in P}{\sum }\left|g\left(s_{1},p\right)-g\left(s_{2},p\right)\right|$$ (1)

where *P* is the set of all SNPs and g (*s*, *p*) is the genotype of sample *s* at position *p*. It is possible that either, or both, of *g* (*s*_1_, *p*) or *g* (*s*_2_, *p*) are unknown, in which case this SNP is ignored in the summation. To account for the fact that the distance between samples may therefore sum across a different number of samples, we include a normalizing term, 1/*n*, where *n* is the number of SNPs actually included in the summation. Distance measures other than the taxicab distance could be used, the most obvious alternative being the Euclidean distance. Using the Euclidean distance rather than the taxicab distance did not noticeably change performance (data not shown).

The algorithm proceeds by picking the *k* nearest neighbors to *s_i_* that have a known genotype at position *p* and then imputing the genotype, *g*_i_ (*p_j_*, *s*_i_), as a weighted modal average of these genotypes (Schwender 2012). That is:

$$g_{i}\left(s_{i},p_{j}\right)=\underset{a\in \left\{0,1,2\right\}}{\arg\max}\underset{s\in N}{\sum }\frac{1}{d_{n}\left(s_{i},s\right)}I\left(g\left(s,p_{j}\right)=a\right)$$ (2)

where *N* is the set of *k* samples nearest *s_i_* , which have a known genotype at SNP $p_{j}.\;I\left(g\left(s,p_{j}\right)=a\right)$ is an indicator function that takes the value 1 if *g* (*s*, *p_j_*) = *a* and 0 otherwise.

Standard *k*-nearest neighbor relies on the assumption that the most similar samples across the whole genome will be the best samples with which to impute any genotype. However, we reasoned that the best samples to use for imputation are those that share an evolutionary history at the SNP to be imputed, and that these samples may not be the most similar genome-wide. Genotype imputation methods like Beagle and fastPHASE use a similar reasoning: they use information from neighboring SNPs because these SNPs likely share a history with the SNP to be imputed due to physical linkage. Beagle and fastPHASE rely, however, on ordered markers and sufficiently dense genotype data to enable haplotype reconstruction. In the absence of a known marker order, we reasoned that the most informative samples for imputation may be those that are most similar at SNPs that are highly correlated with the SNP to be imputed. These highly correlated SNPs may be physically linked to the SNP of interest, but they may also be correlated without being physically linked. Regardless of the reason for the correlation, we reasoned that these SNPs would be most informative for choosing the nearest neighbors with which to perform imputation.

We therefore introduce LD *k*-nearest neighbor imputation (LD-kNNi), where only the *l* SNPs most in LD with the SNP to be imputed are used to determine the nearest neighbor and the weightings to be used when imputing. The “LD” in our algorithm name, LD-kNNi, does not necessarily refer to physical linkage but rather to the correlation between any two SNPs in the data.

Thus, Equation 1 becomes:

$$d_{l}\left(s_{1},s_{2}\right)=\text{c}+\frac{1}{n}\underset{p\in L\left(p_{i}\right)}{\sum }\left|g\left(s_{1},p\right)-g\left(s_{2},p\right)\right|$$ (3)

where *L*(*p_i_*) is the set of *l* SNPs in strongest LD with the SNP to be imputed. The algorithm then continues as for kNNi with one minor exception. As we are now using a smaller number of SNPs to calculate distance, it is possible that there could be no genetic difference between a pair of samples. To avoid a distance of zero, which would result in Equation 2 being undefined, we add a constant to the standard taxicab distance. We set this constant to one as adding different values has little effect on imputation performance (Supporting Information, Table S1 and File S1).

We implement LD-kNNi in our program LinkImpute (available from http://www.cultivatingdiversity.org/software under the GPL version 3 license). LinkImpute allows parameter values (*k* for kNNi, *k* and *l* for LD-kNNi) to be either fixed or optimized. To optimize the parameter values, LinkImpute randomly samples 10,000 known genotypes. For each set of parameter values, each one of these genotypes is masked and then imputed to calculate accuracy. LinkImpute optimizes the parameters by assuming the parameter space is unimodal, bounding the search space and then searching the space for the optimal values.

When comparing different imputation methods on the apple dataset, we used manually optimized parameter values (*k* for kNNi, *k* and *l* for LD-kNNi). For kNNi, *k* = 8 was chosen. For LD-kNNi, *k* = 5 and *l* = 20 (Figure S1). When comparing imputation programs on different datasets, we allowed LinkImpute to choose optimal parameters.

### Data

We collected GBS data from a collection of 1995 accessions from the genus *Malus* from the US Department of Agriculture apple germplasm repository in Geneva, NY. The samples were processed with two different restriction enzymes (ApeKI, *Pst*I/EcoT22I) in separate GBS libraries and were sequenced using Illumina Hi-Sequation 2000 technology. Genotypes were called using a custom GBS pipeline described in Gardner *et al.* (2014). Briefly, 100-bp reads generated from both enzymes were aligned to the *Malus domestica* reference genome version 1.0 (Velasco *et al.* 2010) using the default parameters in BWA (Li and Durbin 2009). Genotypes were called using GATK (McKenna *et al.* 2010) with a minimum of eight reads supporting each genotype. The final genotype matrix was filtered to contain only samples from the domesticated apple, *Malus domestica*, and ≤20% missing data per SNP and per sample. SNPs with a minor allele frequency (MAF) of <0.01 were then discarded. Finally, the data were pruned to exclude clonal relationships: if two or more samples had IBD >0.9, they were considered clones and the sample with the least amount of missing data from the group was retained. This resulted in a dataset of 711 samples and 8404 SNPs.

To test the accuracy of our imputation method we created a “masked” dataset by setting 10,000 random genotypes to missing. This created “truth known” genotypes to which our imputed genotype calls were compared. We limited our testing to 10,000 masked genotypes, which represents 0.17% of the genotype matrix, in order to maintain a dataset with a reasonable amount of missing data while providing enough masked genotypes to be able to estimate imputation accuracy.

Biased allele frequency in imputed data has been shown to affect downstream analyses (Han *et al.* 2014). To determine how well each imputation method estimates allele frequencies, we filtered the genotype matrix to contain no missing data. This resulted in a matrix containing 1001 SNPs from 459 samples (Figure S2). We masked and then imputed 20% (91,952 genotypes) of the genotypes at random and compared the allele frequency estimates from the imputed data to the allele frequency estimates from the complete genotype matrix. As most imputation methods make use of other SNPs to aid imputation, we imputed using all 8404 SNPs in the dataset so as to provide more information to these methods. We then restrict our analysis to the 1001 complete SNPs.

We also tested the performance of our method on genome-wide SNP data from maize and grape. The maize data were downloaded from the International Maize and Wheat Improvement Center (Hearne *et al.* 2014). We reduced the data to biallelic SNPs with <20% missing data and a MAF >1% and then discarded samples with >20% missing data. This resulted in 43,696 SNPs from 4300 samples.

To generate the grape dataset we collected GBS data from a collection of diverse samples from the genus *Vitis* including commercial *Vitis vinifera* varieties, hybrids and wild accessions from the USDA grape germplasm collection. The samples were processed with two different restriction enzymes (*Hin*dIII/BfaI, *Hin*dIII/*Mse*I) and were sequenced using Illumina Hi-Sequation 2000 technology. We then used the 12X grape reference genome (Jaillon *et al.* 2007; Adam-Blondon *et al.* 2011) and the Tassel / BWA version 4 pipeline to generate a genotype matrix (Li and Durbin 2009; Glaubitz *et al.* 2014). Default parameters were used at each stage except for the SNP output stage where we filtered for biallelic SNPs. We then removed any genotypes with fewer than eight supporting reads using vcftools (Danecek *et al.* 2011). Using PLINK (Purcell *et al.* 2007), we removed SNPs with >20% missing data before removing samples with >20% missing data. We then removed SNPs with excess heterozygosity (failed a Hardy−Weinberg equilibrium test with a p-value < 0.001) and finally SNPs with a MAF < 0.01. This created a dataset of 8506 SNPs and 77 samples.

### Other imputation methods

We compared LD-kNNi, as implemented in LinkImpute, with several other imputation methods and programs that do not require a reference panel:

#### Generic imputation methods:

1. *Mode:* The modal value of all other samples’ genotypes at the SNP of interest. Implemented in LinkImpute.
2. k *Nearest Neighbor:* As described above and implemented in LinkImpute.
3. *MF:* As implemented in the R package MissForest (version 4.6-10) with maxiter set to 10 and ntree to 100 and all other parameters set to default values. MF could not be run on the entire genome at once because the run time was prohibitive. Instead it was run one chromosome at a time (Stekhoven and Bühlmann 2012).

#### Genotype-specific methods:

4. *Beagle:* Version r1230 using default settings (Browning and Browning 2007).
5. *fastPHASE:* Version 1.4.0 using default settings except K (the number of clusters) is set to 200 (Scheet and Stephens 2006).

### Data availability

The datasets used in this study are available in Supporting Information File S1. LinkImpute is available from http://www.cultivatingdiversity.org/software under the GPL version 3 license.

## Results

### Imputation accuracy

We first compared the accuracy of imputation by using the large apple dataset where we randomly masked 10,000 genotypes. We compared the imputed genotypes with the actual genotypes and calculated two measures of accuracy. Genotype error is the proportion of genotypes called incorrectly and allele error is the proportion of alleles called incorrectly. The methods rank similarly for both measures (Table 1), although allele error is approximately half of genotype error. This is to be expected, because all methods that are likely to impute one allele correctly are unlikely to impute both alleles incorrectly.

**Table 1:** Performance of the different imputation methods on the apple dataset

Method	Genotype Error	Allele Error	Run Time, sec
Mode	23.0%	12.4%	*^a^*
kNNi*^b^*	20.6%	10.8%	18
MF	9.9%	5.1%	40,107
fastPHASE	7.7%	3.9%	52,399
Beagle	7.6%	3.9%	424
LD-kNNi*^c^*	7.4%	3.9%	104

kNNi, *k*-nearest neighbors imputation; LD-kNNi, linkage disequilibrium *k*-nearest neighbors imputation.

a Run time was under a second.

b Using a fixed value of *k* = 8.

c Using fixed values of *k* = 5 and *l* = 20.

**Figure 1:**
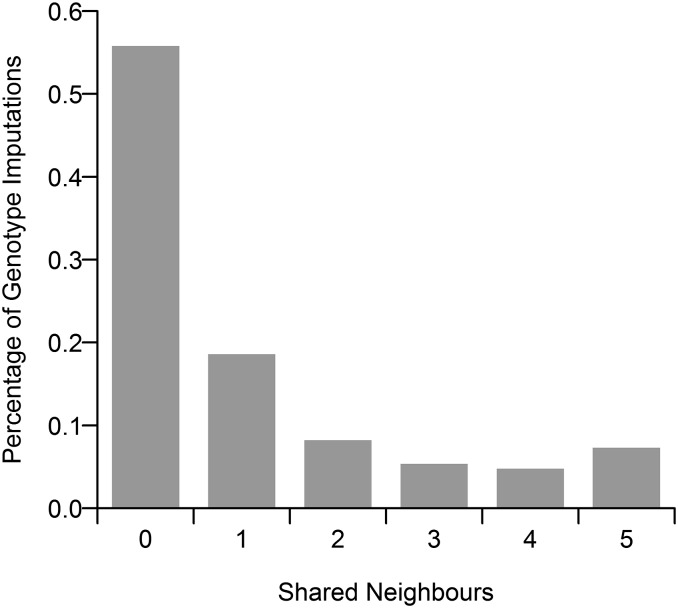
The number of shared neighbors between the *k*-nearest neighbors imputation (kNNi) and linkage disequilibrium *k*-nearest neighbors imputation (LD-kNNi) methods. The value of *l* was set to 5 for both methods.

Our results show that LD-kNNi performs slightly better than Beagle and fastPHASE, which have the greatest accuracy of all the other methods tested (Table 1). MF performs noticeably worse than these methods, although this may, in part, be due to having imputed on a per chromosome basis. kNNi performs significantly worse than any of these methods, only slightly out-performing Mode imputation. We investigated the difference between LD-kNNi and kNNi further by computing, for each imputed genotype, the number of neighbors that are shared between the kNNi and LD-kNNi methods, using *k* = 5 in both cases. We found that in 56% of imputations the two methods share no neighbors (Figure 1). This finding suggests that in many cases kNNi is imputed using samples that, although similar across the whole genome, may not be informative for the SNP we are imputing. Figure S3 further supports this hypothesis by measuring the average distance, using the LD-kNNi methodology (*d_l_*, Equation 3), from the sample to be imputed to the neighbors being used in the imputation. This shows that the average distance, again using *k* = 5 in both cases, is much greater in the case of kNNi (average distance: 6.4) than LD-kNNi (average distance: 1.8).

**Figure 2:**
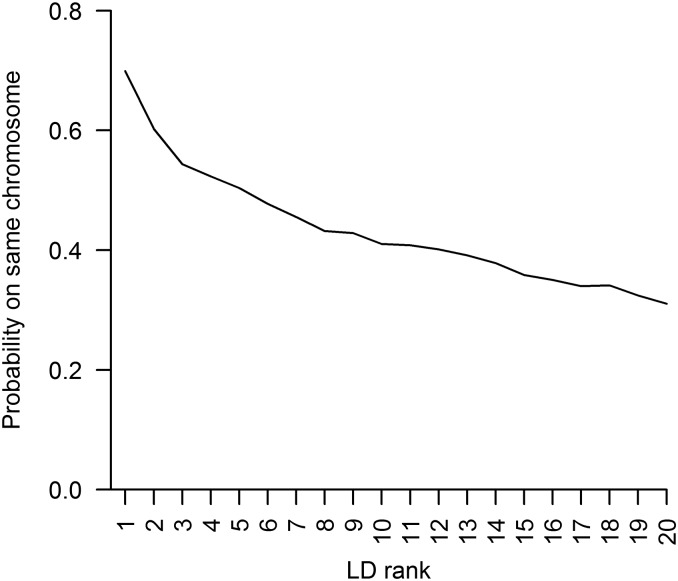
The probability of a single-nucleotide polymorphism (SNP) being on the same chromosome as the imputed SNP as a function of linkage disequilibrium (LD) with the imputed SNP. SNPs are ranked according to LD, with the SNP most in LD with the imputed SNP ranked one.

Unsurprisingly, we found that the performance of LD-kNNi is dependent on the level of LD between the SNP to be imputed and the SNPs used to find the nearest neighbors. Where the average LD between the SNPs used and the imputed SNP is high, the imputation error is lower (Figure S4). Although the apple reference genome is not used in LD-kNNi, we exploited it to investigate how often our nearest neighbor calculations used SNPs from chromosomes other than the chromosome on which the imputed SNP is located. To do this, we calculated the probability of being on the same chromosome as the imputed SNP for the 20 SNPs in greatest LD with the imputed SNP. Figure 2 shows that for the SNP with the highest LD, there is a probability of 0.7 of being on the same chromosome and that this drops off to 0.31 for the 20th-ranked SNP.

**Figure 3:**
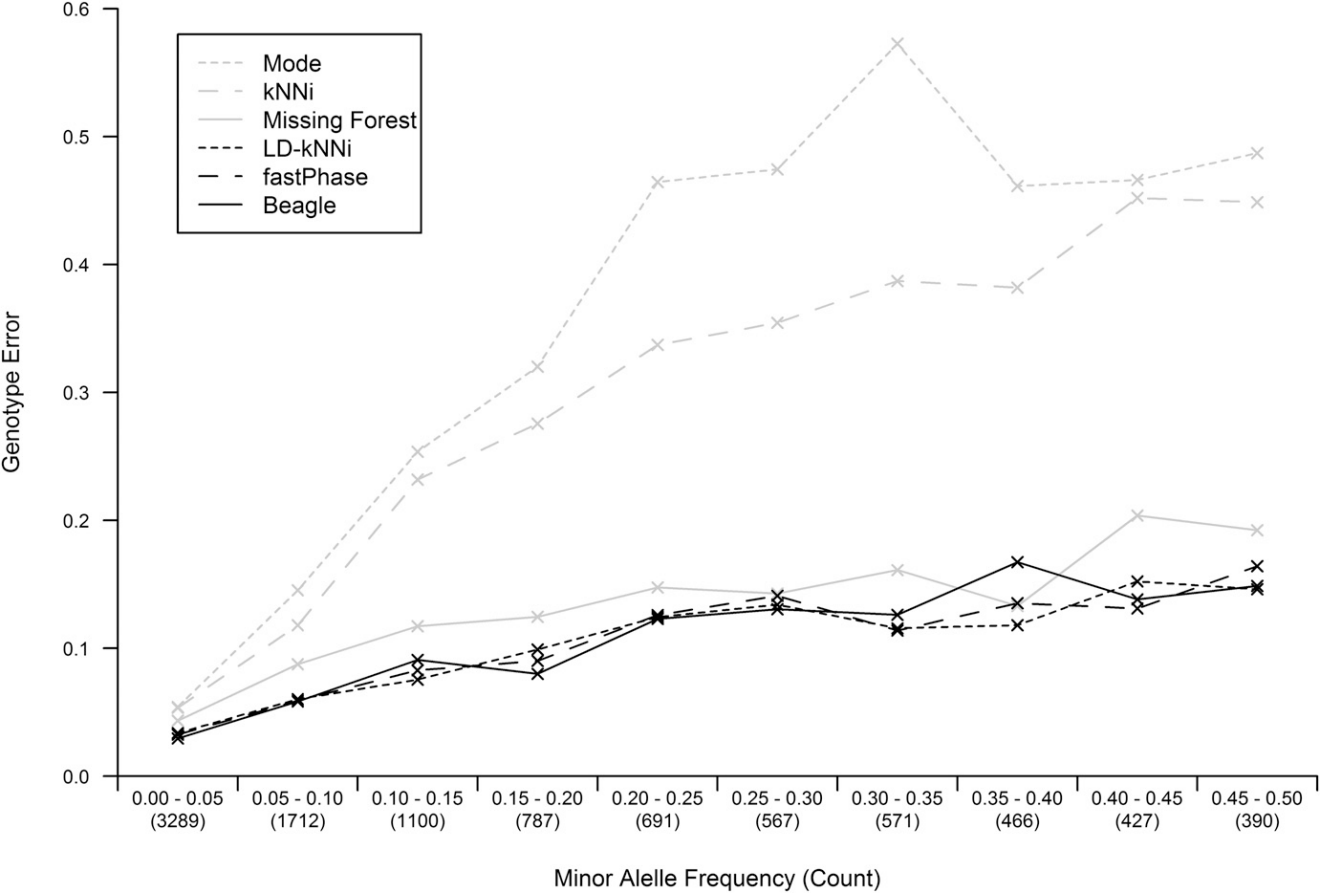
Imputation accuracy as a function of the minor allele frequency (MAF) of the imputed SNP for each of the six imputation methods. MAF is binned in 5% bins and the number of SNPs in each bin is shown in parentheses. kNNi, *k*-nearest neighbors imputation; LD-kNNi, linkage disequilibrium *k*-nearest neighbors imputation.

We investigated the performance of the different imputation methods based on the MAF of the imputed SNPs. Figure 3 shows the genotype error rate of the different methods stratified by MAF. While the error rate noticeably increased with MAF for Mode and kNNi, the increase is small for the other four methods.

### Run time

Comparing the run time of the various imputation methods (Table 1), we note that both MF and fastPHASE took significantly longer than any of the other methods: these two methods take on the order of 10 hr compared with only a few minutes or less for the other methods. Further analysis of the run time suggests that, as both the number of samples and SNPs increases, LD-kNNi will continue to have a shorter run time than Beagle (Figure S7 and Figure S8).

Comparing the performance of LinkImpute and Beagle on multiple datasets (Table 2) we note that LinkImpute has a similar run-time to Beagle on all three datasets while achieving slightly better accuracy.

**Table 2:** Performance of LinkImpute and Beagle on different datasets

Dataset	Number of SNPs	Number of Samples	Genotype Error		Run Time, sec	
			LinkImpute*^a^*	Beagle	LinkImpute*^a^*	Beagle
Apple	8404	711	7.4%	7.6%	104	424
Maize	43,696	4300	18.1%	18.7%	7608	16,585
Grape	8506	77	9.5%	11.0%	28	16

SNP, single-nucleotide polymorphism.

a Using the LD-kNNi option and optimized values of *k* and *l*

### Accuracy of allele frequency estimation

**Figure 4:**
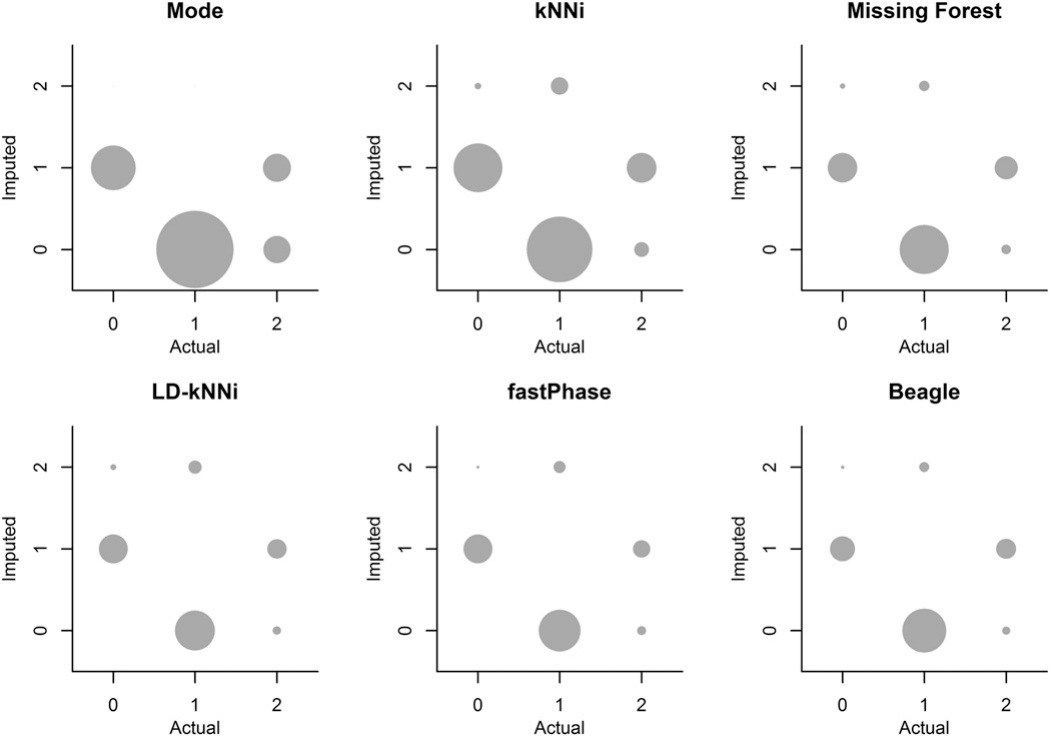
Bubble plots of the actual and imputed genotypes for each of the 10,000 masked genotypes using each of the six imputation methods. Bubbles are not shown for the correctly imputed cases. The size of the bubbles is proportional to the frequency of observations in that category. kNNi, *k*-nearest neighbors imputation; LD-kNNi, linkage disequilibrium *k*-nearest neighbors imputation.

Figure 4 shows a bubble plot of actual and incorrectly imputed genotypes for each of the six imputation methods. This shows that all six methods have a bias toward imputing the major allele. This allele bias is pronounced for Mode and kNNi and is less severe for the other methods.

**Figure 5:**
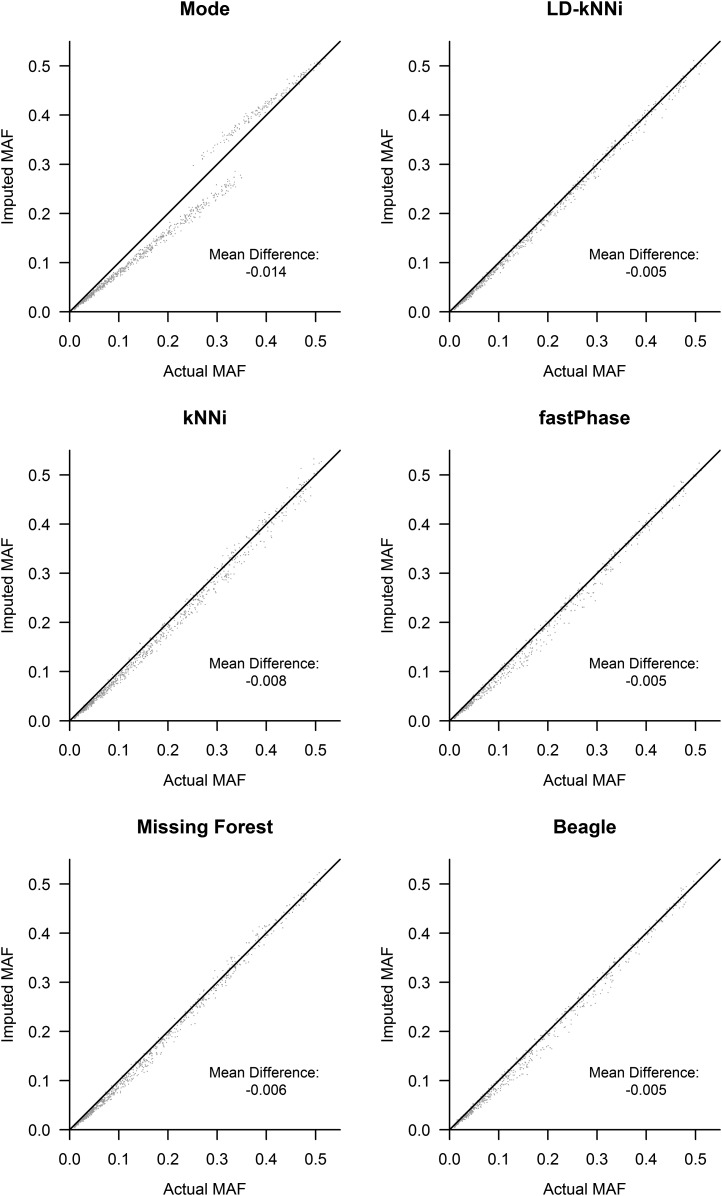
Minor allele frequency (MAF) computed by the use of actual and imputed genotypes for each of the six imputation methods. kNNi, *k*-nearest neighbors imputation; LD-kNNi, linkage disequilibrium *k*-nearest neighbors imputation.

The allele bias observed in Figure 4 is expected to affect allele frequency estimation. We investigated this further by using our smaller dataset. For each of the six methods, we calculated the MAF across the 1001 SNPs without missing genotype data using both the observed genotypes and the imputed genotypes. Figure 5 shows that every imputation method biases the MAF downward. This finding is consistent with our observation of allele bias in Figure 4. The resulting bias is least pronounced for genotype specific methods, which all bias the MAF downward by 0.5% as opposed to a minimum of 0.6% for any of the other methods.

Figure 5 shows the tendency for the MAF to be underestimated when calculated using an imputed dataset no matter what imputation method is used. In addition, LD-kNNi outperforms every other method in estimating MAF: the points cluster much closer to the line for LD-kNNi than for any of the other methods. Moreover, LD-kNNi’s most extreme deviation (3.8%) from the observed MAF is lower than any of the other tested methods (Figure S5). The two groups in the Mode plot are caused by the two different modal values for SNPs (0 or 1). The bottom left group is where the modal value is 0, the top right group is where it is 1 (Figure S6).

## Discussion

LD-kNNi performs well compared with the most commonly used imputation methods. On our apple dataset it results in both superior imputation accuracy (Table 1) and more accurate allele frequency estimates (Figure 4 and Figure 5). Accuracy results on the two other tested datasets are similar, and the results presented here suggest that performance should be comparable on other similar datasets. In particular, Figure 3 suggests that the MAF distribution should have little effect on the relative performance of LD-kNNi.

The run time of LinkImpute also compares favorably with existing methods. Only two of the methods studied here have both high accuracy and reasonable run times, namely Beagle and the LD-kNNi option of LinkImpute. Of these, our method is slightly faster. In addition, as the number of samples and SNPs increases, LD-kNNi is expected to outperform the other methods (Figure S7 and Figure S8), which is particularly noteworthy because increasing sample size is critical to augmenting the statistical power of GWA studies (Spencer *et al.* 2009).

A recently developed imputation algorithm was designed for heterozygous species without a reference genome and was applied to raspberry (genus *Rubus*; Ward *et al.* 2013). However, this method applies only to biparental populations and relies on the construction of a genetic map. The primary advantage of LD-kNNi over existing methods is that it does not rely on ordered markers and can be applied to diverse and heterozygous populations (Figure S9), not just biparental crosses. Although we called SNPs using the apple reference genome, LD-kNNi makes no use of this information during imputation. Indeed Figure 2 shows that in many cases our algorithm is using information from SNPs that are not on the same chromosome as the imputed SNP. It is worth noting that linkage group assignments from apple F1 populations conflict with reference genome locations for 14–18% of SNPs (Antanaviciute *et al.* 2012; Gardner *et al.* 2014). It is therefore likely that a significant number of sequences are anchored incorrectly in the version of the apple genome used here. Thus, the values in Figure 2 may be upward biased. Nevertheless, LD-kNNi clearly often makes use of information from SNPs on other chromosomes and the quality of the apple reference genome has no effect on its performance.

We demonstrated that the performance of LD-kNNi improves as the LD between the imputed SNPs and the SNPs used to find the nearest neighbors increases (Figure S4). This suggests that, as the SNP density of a dataset increases and more SNPs are in LD with one another, one can expect improvements in the imputation accuracy of LD-kNNi. One way of obtaining more SNPs would be to allow greater levels of missing genotypes, although the increase in missing data are likely to have a negative effect on imputation accuracy. Whether this negative effect is offset by the positive effect of increased SNP density is an area that warrants further study.

Like most other imputation methods, LinkImpute is applied to a table of genotypes that have been called by a genotype calling algorithm. In many cases, a genotype without sufficient sequence coverage is set to missing in the table even though it has several supporting sequence reads from the original data source. In such cases, the information from those reads is lost and remains unused during imputation. By including the information from these reads during imputation, we are likely to improve imputation performance. In turn, this should enable greater confidence genotype calls from lower read depths thereby significantly increasing the total number of genotypes called. Moreover, incorporating imputation and SNP calling in this manner should help improve genotyping error rates, especially in cases of low read depth. This is an active area of research and future improvements are expected to increase both genotype quality and quantity.

Our results suggest that LD-kNNi produces more accurate allele frequency estimates at the cost of a slight decrease in imputation accuracy. Biased allele frequencies are known to adversely affect downstream analyses (Han *et al.* 2014), whereas increased imputation accuracy does not always lead to improved phenotype prediction (Rutkoski *et al.* 2013). For many studies, an imputation method with less bias in allele frequency estimation, such as LD-kNNi, may therefore be preferable to a method with slightly increased accuracy. It is worth noting that, in cases where one is only interested in the MAF, one can simply estimate it from the nonmissing genotypes. We show that such an estimate is indeed unbiased and that it is more accurate than estimating MAF after imputation (Figure S5 and Figure S10). The relationship between imputation accuracy, allele frequency bias and their effects on downstream analyses warrants further investigation.

Genotype imputation is a crucial step in many genomic studies as all existing genotyping methods result in some missing data. Most imputation algorithms rely on physical or genetic maps, either directly or in the generation of ordered SNPs, and are not suitable for use in non-model organisms with poor or underdeveloped genomic resources. Our novel genotype imputation method, LD-kNNi, does not rely on physical or genetic maps and imputes genotypes as accurately as the best existing methods that require ordered markers. In addition, it is fast and outperforms other methods in its ability to accurately estimate allele frequencies. Thus, LinkImpute is a valuable tool for improving genome-wide analyses in nonmodel organisms, especially for GWA and GS in highly diverse and heterozygous organisms.

## 

## Supplementary Material

Supporting Information
