# Supplementary material for: LinkImpute: Fast and Accurate Genotype Imputation for Nonmodel Organisms
Source: G3 (Bethesda). 2015 Sep 15;5(11):2383–90. doi: 10.1534/g3.115.021667 (PMC4632058; doi:10.1534/g3.115.021667)
Supplement: Supporting Information [file supp_5_11_2383__index.html]

LinkImpute: Fast and Accurate Genotype Imputation for Nonmodel Organisms — Supporting Information 

# LinkImpute: Fast and Accurate Genotype Imputation for Nonmodel Organisms

## Supporting Information for Money *et al.*, 2015

**Files in this Data Supplement:**

- Supporting Information - Figures S1-S10, Table S1, and File S1 (PDF, 1 MB)
- Figure S1 - Heat map showing genotype imputation error, using LD-kNNi, for different numbers of neighbors (*k*) and number of SNPs used (*l*). (PDF, 256 KB)
- Figure S2 - Number of samples remaining as a function of the number of SNPs without missing data. (PDF, 213 KB)
- Figure S3 - Average distance to neighboring samples using kNNi and LD-kNNi. (PDF, 284 KB)
- Figure S4 - Imputation accuracy as a function of LD between the imputed SNP and the SNPs used for imputation. (PDF, 208 KB)
- Figure S5 - Difference in MAF computed using actual and imputed genotypes for each of the imputation methods. (PDF, 237 KB)
- Figure S6 - Actual MAF compared to MAF estimated with imputed genotypes for the Mode method. (PDF, 228 KB)
- Figure S7 - Run time as a function of the number of SNPs. (PDF, 244 KB)
- Figure S8 - Run time as a function of sample size. (PDF, 238 KB)
- Figure S9 - Box plots of the average heterozygosity by sample for each of our three datasets. (PDF, 161 KB)
- Figure S10 - MAF calculated using the actual MAF (calculated before masking) to the MAF calculated including missing data (after masking). (PDF, 215 KB)
- Table S1 - The effect of the constant, *c*, in Equation 3 on accuracy. (PDF, 121 KB)
- File S1 - Apple and grape datasets used in this study. Also included are instructions on how to recreate the maize dataset. (.zip, 2 MB)
