# Supplementary material for: LinkImpute: Fast and Accurate Genotype Imputation for Nonmodel Organisms
Source: G3 (Bethesda). 2015 Sep 15;5(11):2383–90. doi: 10.1534/g3.115.021667 (PMC4632058; doi:10.1534/g3.115.021667)
Supplement: Supporting Information [file supp_g3.115.021667_021667SI.pdf]

G3·Genes | Genomes | Genetics

LinkImpute: fast and accurate genotype imputation for non-model organisms

Daniel Money*[,1], Kyle Gardner*, Heidi Schwaninger[§], Gan-Yuan Zhong[§], Sean Myles*

* Department of Plant and Animal Sciences, Faculty of Agriculture, Dalhousie University, Truro, Nova Scotia, B2N 5E3, Canada.

[§] USDA-ARS Plant Genetic Resources Unit, 630 W. North St., Geneva, NY 14456

[1] Corresponding Author: Department of Plant and Animal Sciences, Faculty of Agriculture, Dalhousie University, Truro, Nova Scotia, B2N 5E3, Canada. E-mail: daniel.money@dal.ca

DOI: 10.1534/g3.115.021667

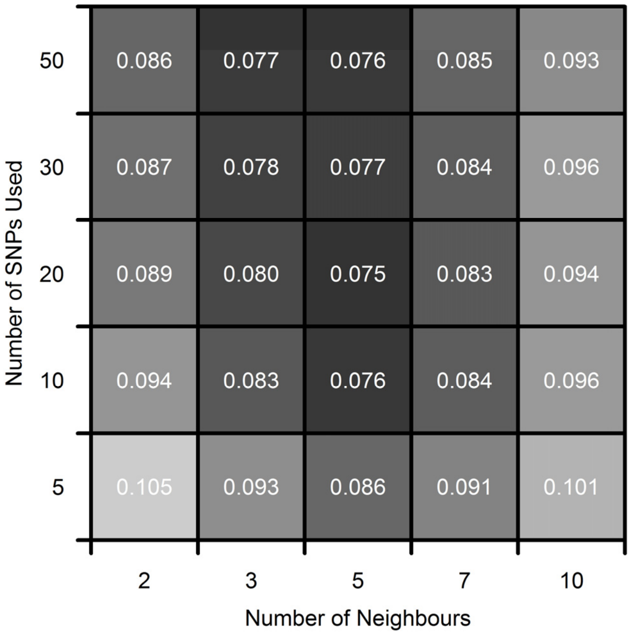

Figure S1: Heat map showing genotype imputation error, using LD-kNNi, for different numbers of neighbours (*k*) and number of SNPs used (*l*). Results are shown for the apple dataset and for selected values of *k* and *l*. Darker boxes indicate less error. We chose *k* = 5 and *l* = 20 for this study.

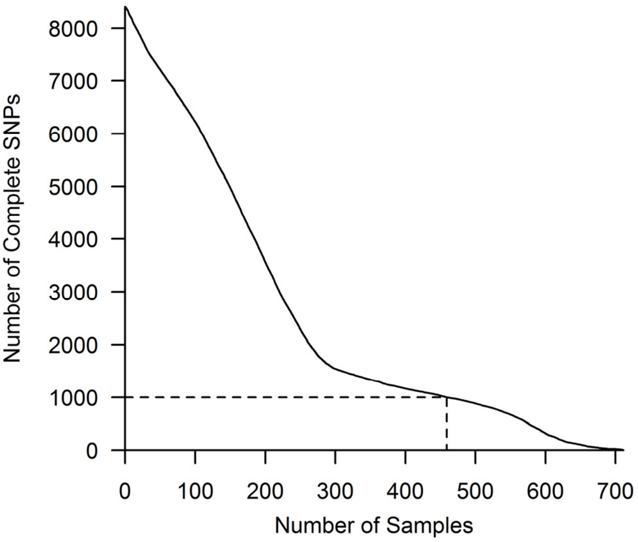

Figure S2: Number of samples remaining as a function of the number of SNPs without missing data. By removing samples we increase the number of SNPs without missing data. We chose the largest number of samples that resulted in 1000 or more SNPs without missing data. This resulted in 459 samples and 1001 SNPs.

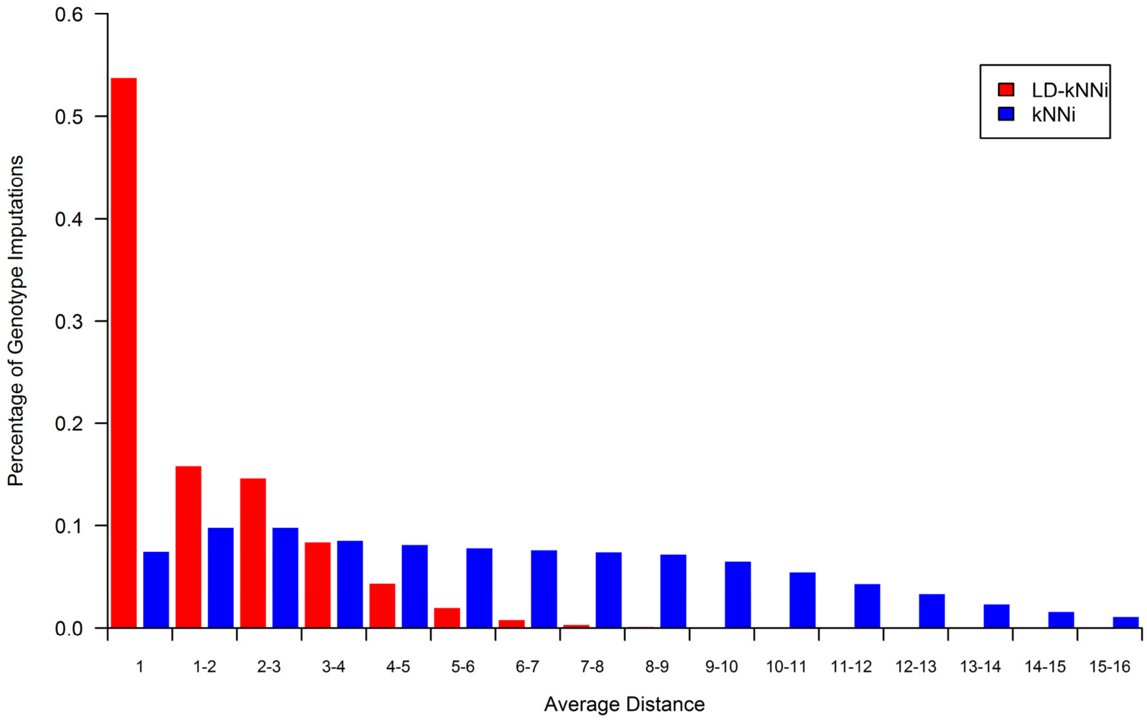

Figure S3 Average distance to neighbouring samples using kNNi and LD-kNNi. To allow comparison, the d_l distance measure (Equation 3) is used in each case and both methods use k = 5. Exactly one is shown as a separate bar due its high frequency. The average distance to neighbouring samples is far lower for LD-kNNi than for kNNi.

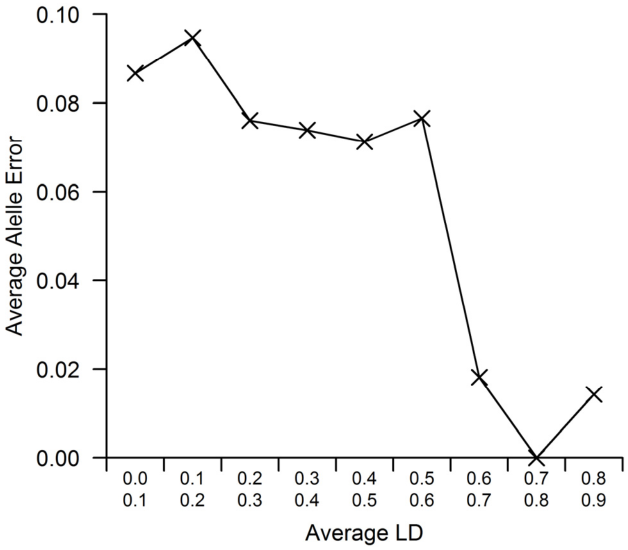

Figure S4 Imputation accuracy as a function of LD between the imputed SNP and the SNPs used for imputation. LD is binned in bins of size 0.1. The increase between bins 0.7-0.8 and 0.8-0.9 is caused by the low number of SNPs in each bin (60 and 70 respectively) and the low number of wrongly called imputations in each case (0 and 1 respectively).

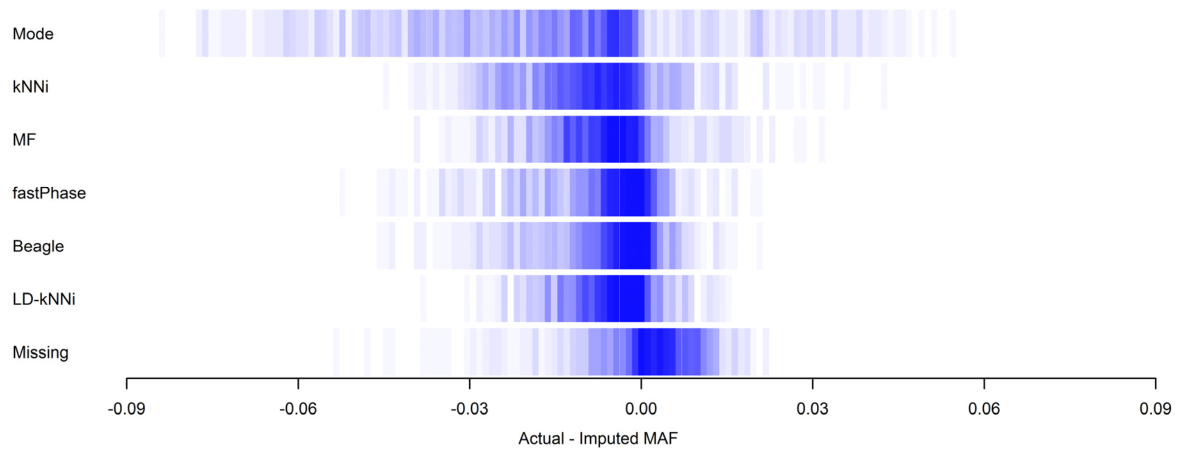

Figure S5 Difference in MAF computed using actual and imputed genotypes for each of the imputation methods. The darker the line, the more SNPs show this difference in allele frequency. “Missing” compares the actual MAF (calculated before masking) to the MAF calculated including missing data (after masking).

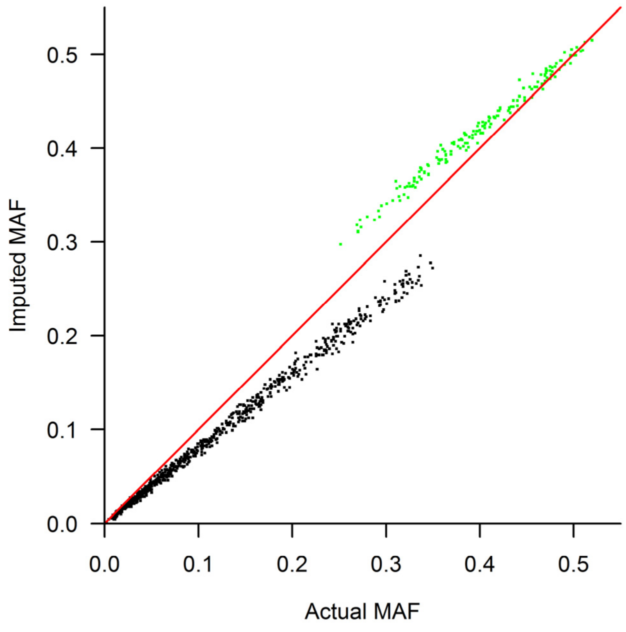

Figure S6 Actual MAF compared to MAF estimated with imputed genotypes for the Mode method. Points are colored by the modal value of the SNP being imputed, black for 0 and green for 1. The Mode method generally overestimates MAF when imputing a 1 (heterozygote) and underestimates MAF when imputing a 0 (homozygous major allele).

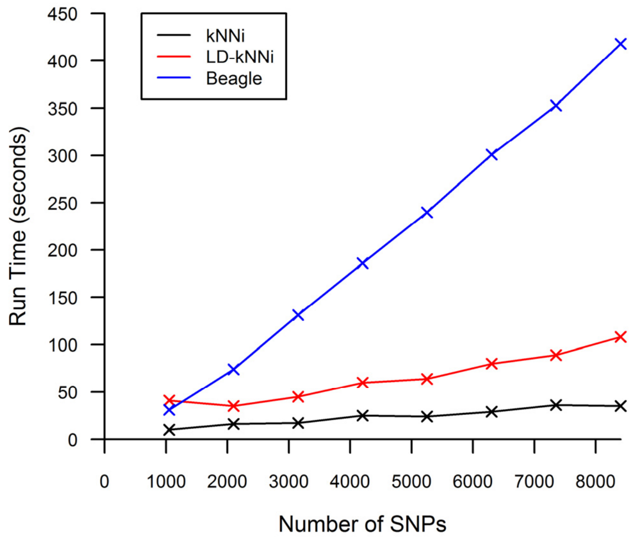

Figure S7 Run time as a function of the number of SNPs. SNPs were removed at random from our larger dataset to produce smaller sets of SNPs. Random Forest and fastPhase are not shown due to their long run times. Mode is not shown as its run time was under one second on the full data set.

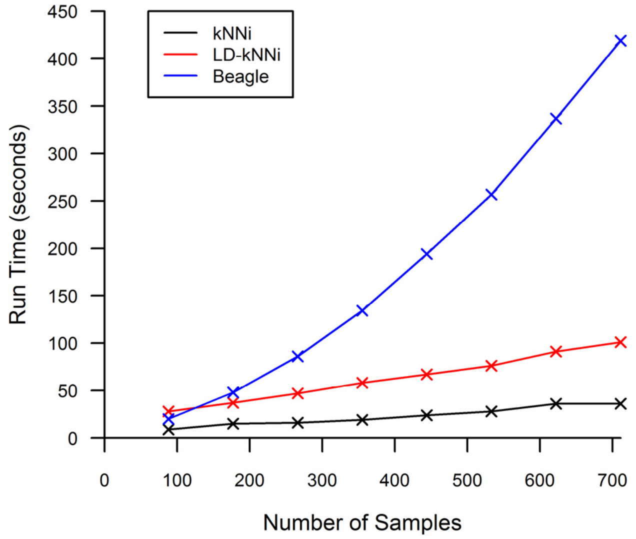

Figure S8 Run time as a function of sample size. Samples were removed at random from our larger dataset to produce smaller sample sizes. Random Forest and fastPhase are not shown due to their long run times. Mode is not shown as its run time was under one second on the full data set.

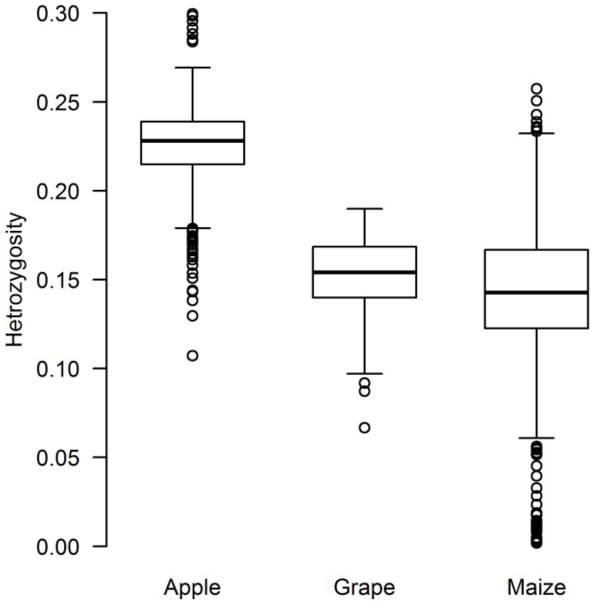

Figure S9 Box plots of the average heterozygosity by sample for each of our three datasets.

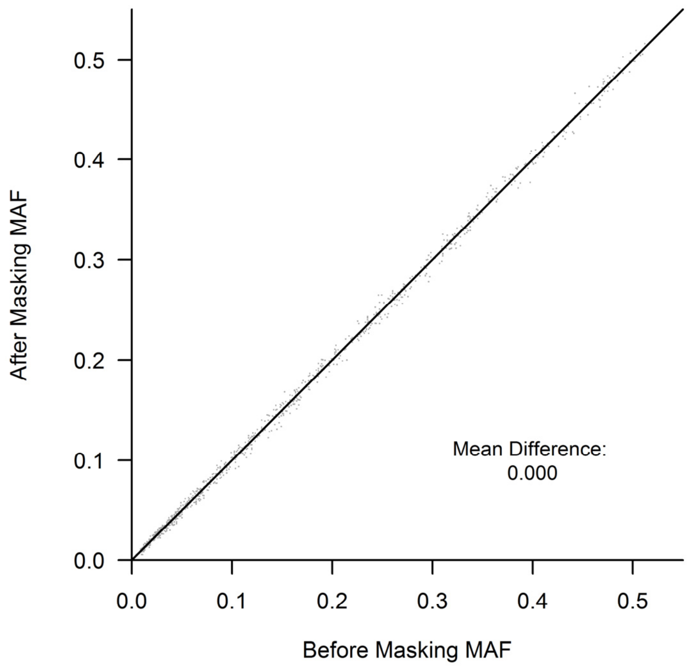

Figure S10 MAF calculated using the actual MAF (calculated before masking) to the MAF calculated including missing data (after masking).

Table S1 The effect of the constant, *c*, in Equation 3 on accuracy. Accuracy is calculated on the Apple dataset using the given values of *k* and *l*.

	k	3			5			10		
	l	10	20	30	10	20	30	10	20	30
Constant	0.1	0.9162	0.923	0.7356	0.9198	0.9242	0.7356	0.9229	0.9228	0.7356
	0.2	0.9162	0.9233	0.7356	0.9198	0.9247	0.7356	0.9236	0.9234	0.7356
	0.5	0.9163	0.9238	0.7356	0.9199	0.9248	0.7356	0.9237	0.9232	0.7356
	1	0.9173	0.9236	0.7356	0.9204	0.9246	0.7356	0.9231	0.9235	0.7356
	2	0.9172	0.9243	0.7356	0.9206	0.9247	0.7356	0.9223	0.9219	0.7356
	5	0.9174	0.9237	0.7356	0.9204	0.9234	0.7356	0.9215	0.9214	0.7356
	10	0.9174	0.9238	0.7356	0.9204	0.9233	0.7356	0.9214	0.9214	0.7356

File S1

Apple and grape datasets used in this study. Also included are instructions on how to recreate the maize dataset.

Available for download as a .zip file at www.g3journal.org/lookup/suppl/doi:10.1534/g3.115.021667/-/DC1
