# Supplementary material for: LinkImpute: Fast and Accurate Genotype Imputation for Nonmodel Organisms
Source: G3 (Bethesda). 2015 Sep 15;5(11):2383–90. doi: 10.1534/g3.115.021667 (PMC4632058; doi:10.1534/g3.115.021667)
Supplement: Supporting Information [file supp_g3.115.021667_FigureS1.pdf]

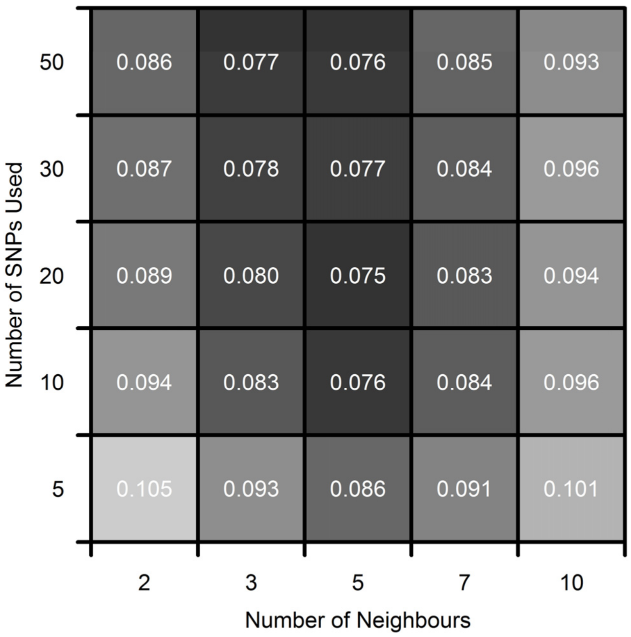

Figure S1: Heat map showing genotype imputation error, using LD-kNNi, for different numbers of neighbours (*k*) and number of SNPs used (*l*). Results are shown for the apple dataset and for selected values of *k* and *l*. Darker boxes indicate less error. We chose *k* = 5 and *l* = 20 for this study.
