# Supplementary material for: LinkImpute: Fast and Accurate Genotype Imputation for Nonmodel Organisms
Source: G3 (Bethesda). 2015 Sep 15;5(11):2383–90. doi: 10.1534/g3.115.021667 (PMC4632058; doi:10.1534/g3.115.021667)
Supplement: Supporting Information [file supp_g3.115.021667_FigureS2.pdf]

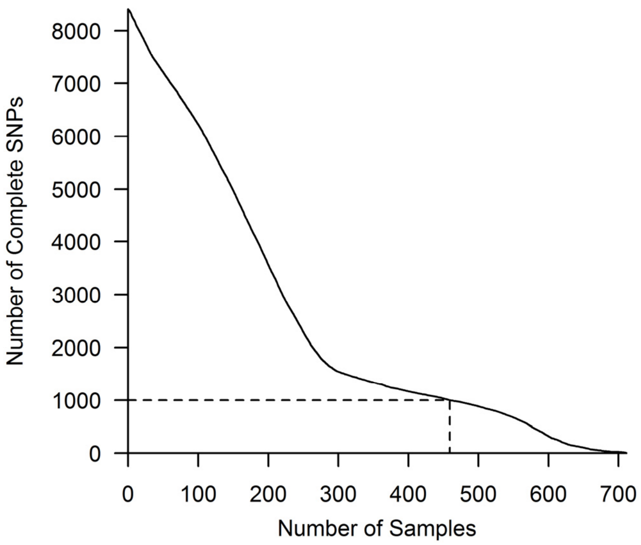

Figure S2: Number of samples remaining as a function of the number of SNPs without missing data. By removing samples we increase the number of SNPs without missing data. We chose the largest number of samples that resulted in 1000 or more SNPs without missing data. This resulted in 459 samples and 1001 SNPs.
