# Supplementary material for: LinkImpute: Fast and Accurate Genotype Imputation for Nonmodel Organisms
Source: G3 (Bethesda). 2015 Sep 15;5(11):2383–90. doi: 10.1534/g3.115.021667 (PMC4632058; doi:10.1534/g3.115.021667)
Supplement: Supporting Information [file supp_g3.115.021667_FigureS3.pdf]

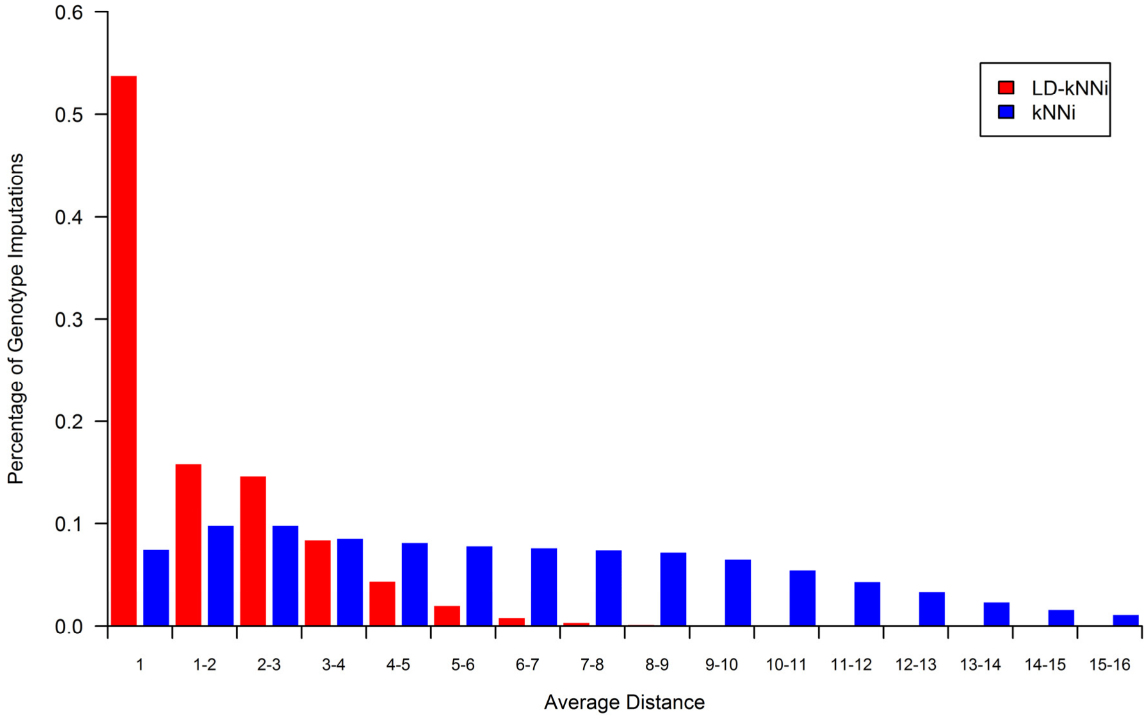

Figure S3 Average distance to neighbouring samples using kNNi and LD-kNNi. To allow comparison, the d_l distance measure (Equation 3) is used in each case and both methods use *k* = 5. Exactly one is shown as a separate bar due its high frequency. The average distance to neighbouring samples is far lower for LD-kNNi than for kNNi.
