# Supplementary material for: LinkImpute: Fast and Accurate Genotype Imputation for Nonmodel Organisms
Source: G3 (Bethesda). 2015 Sep 15;5(11):2383–90. doi: 10.1534/g3.115.021667 (PMC4632058; doi:10.1534/g3.115.021667)
Supplement: Supporting Information [file supp_g3.115.021667_FigureS4.pdf]

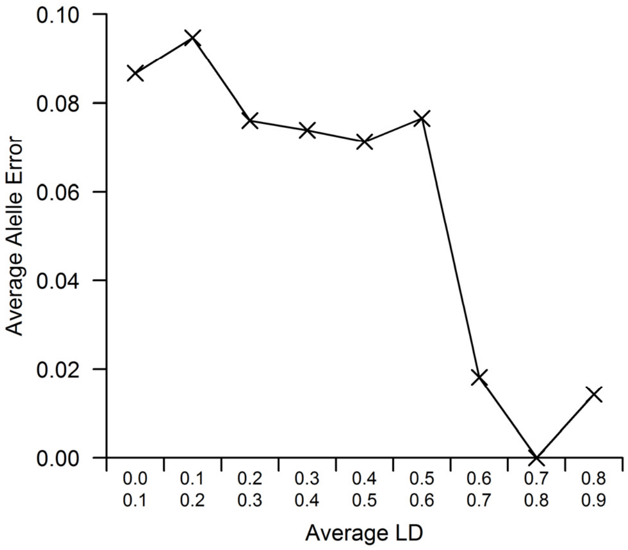

Figure S4 Imputation accuracy as a function of LD between the imputed SNP and the SNPs used for imputation. LD is binned in bins of size 0.1. The increase between bins 0.7-0.8 and 0.8-0.9 is caused by the low number of SNPs in each bin (60 and 70 respectively) and the low number of wrongly called imputations in each case (0 and 1 respectively).
