# Supplementary material for: LinkImpute: Fast and Accurate Genotype Imputation for Nonmodel Organisms
Source: G3 (Bethesda). 2015 Sep 15;5(11):2383–90. doi: 10.1534/g3.115.021667 (PMC4632058; doi:10.1534/g3.115.021667)
Supplement: Supporting Information [file supp_g3.115.021667_FigureS5.pdf]

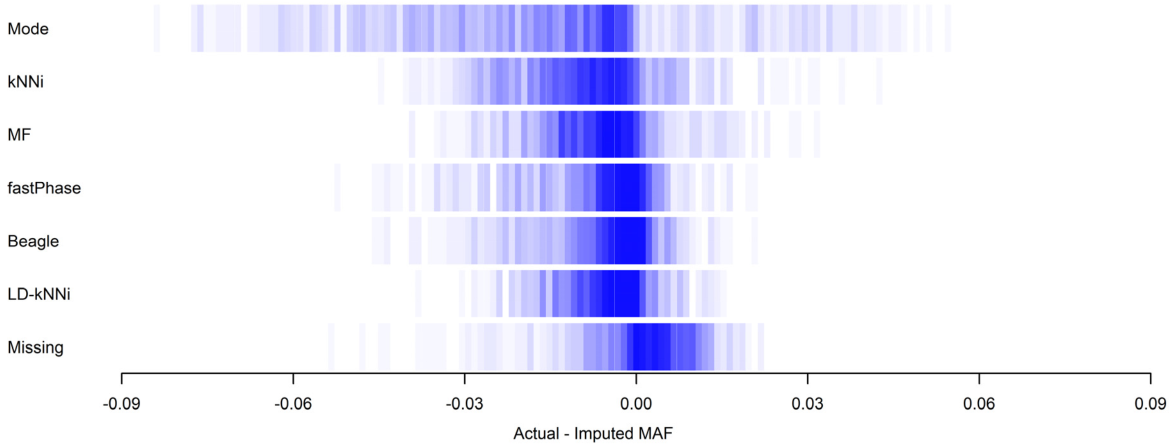

Figure S5 Difference in MAF computed using actual and imputed genotypes for each of the imputation methods. The darker the line, the more SNPs show this difference in allele frequency. “Missing” compares the actual MAF (calculated before masking) to the MAF calculated including missing data (after masking).
