# Supplementary material for: LinkImpute: Fast and Accurate Genotype Imputation for Nonmodel Organisms
Source: G3 (Bethesda). 2015 Sep 15;5(11):2383–90. doi: 10.1534/g3.115.021667 (PMC4632058; doi:10.1534/g3.115.021667)
Supplement: Supporting Information [file supp_g3.115.021667_FigureS6.pdf]

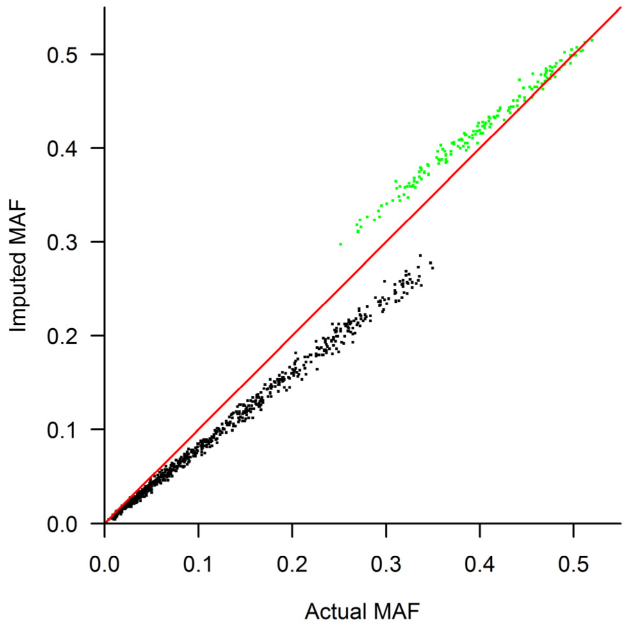

Figure S6 Actual MAF compared to MAF estimated with imputed genotypes for the Mode method. Points are colored by the modal value of the SNP being imputed, black for 0 and green for 1. The Mode method generally overestimates MAF when imputing a 1 (heterozygote) and underestimates MAF when imputing a 0 (homozygous major allele).
