# Supplementary material for: LinkImpute: Fast and Accurate Genotype Imputation for Nonmodel Organisms
Source: G3 (Bethesda). 2015 Sep 15;5(11):2383–90. doi: 10.1534/g3.115.021667 (PMC4632058; doi:10.1534/g3.115.021667)
Supplement: Supporting Information [file supp_g3.115.021667_FigureS7.pdf]

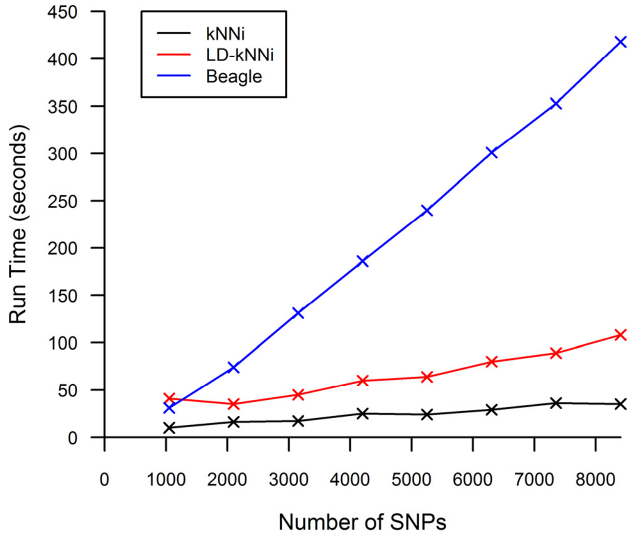

Figure S7 Run time as a function of the number of SNPs. SNPs were removed at random from our larger dataset to produce smaller sets of SNPs. Random Forest and fastPhase are not shown due to their long run times. Mode is not shown as its run time was under one second on the full data set.
