# Supplementary material for: LinkImpute: Fast and Accurate Genotype Imputation for Nonmodel Organisms
Source: G3 (Bethesda). 2015 Sep 15;5(11):2383–90. doi: 10.1534/g3.115.021667 (PMC4632058; doi:10.1534/g3.115.021667)
Supplement: Supporting Information [file supp_g3.115.021667_FigureS8.pdf]

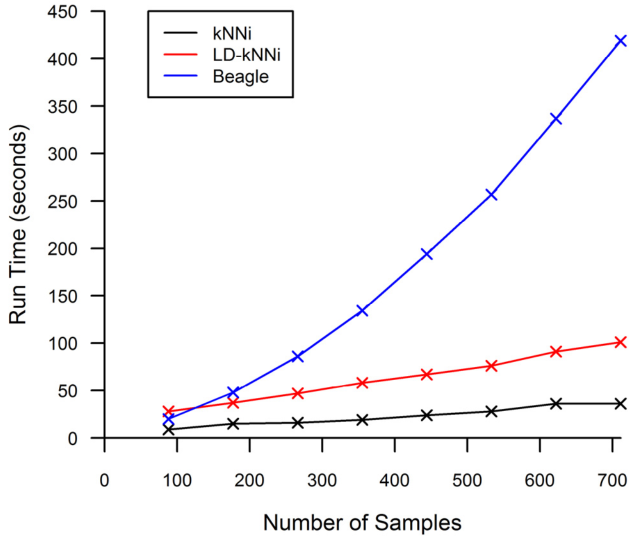

Figure S8 Run time as a function of sample size. Samples were removed at random from our larger dataset to produce smaller sample sizes. Random Forest and fastPhase are not shown due to their long run times. Mode is not shown as its run time was under one second on the full data set.
