# Supplementary figures and images for: LinkImpute: Fast and Accurate Genotype Imputation for Nonmodel Organisms
Source: G3 (Bethesda). 2015 Sep 15;5(11):2383–90. doi: 10.1534/g3.115.021667 (PMC4632058; doi:10.1534/g3.115.021667)

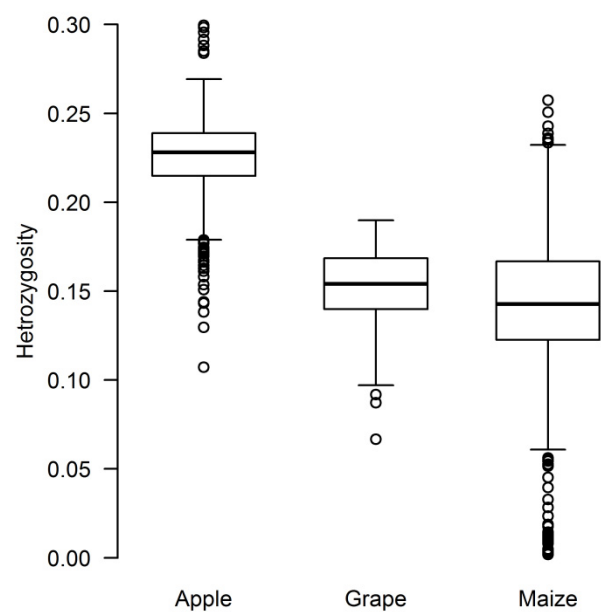

**Figure S9** Box plots of the average heterozygosity by sample for each of our three datasets.

Supplement: Supporting Information [file supp_g3.115.021667_FigureS9.pdf]
