# Supplementary material for: LinkImpute: Fast and Accurate Genotype Imputation for Nonmodel Organisms
Source: G3 (Bethesda). 2015 Sep 15;5(11):2383–90. doi: 10.1534/g3.115.021667 (PMC4632058; doi:10.1534/g3.115.021667)
Supplement: Supporting Information [file supp_g3.115.021667_TableS1.pdf]

Table S1 The effect of the constant, *c*, in Equation 3 on accuracy. Accuracy is calculated on the Apple dataset using the given values of *k* and *l*.

	k	3			5			10		
	l	10	20	30	10	20	30	10	20	30
Constant	0.1	0.9162	0.923	0.7356	0.9198	0.9242	0.7356	0.9229	0.9228	0.7356
	0.2	0.9162	0.9233	0.7356	0.9198	0.9247	0.7356	0.9236	0.9234	0.7356
	0.5	0.9163	0.9238	0.7356	0.9199	0.9248	0.7356	0.9237	0.9232	0.7356
	1	0.9173	0.9236	0.7356	0.9204	0.9246	0.7356	0.9231	0.9235	0.7356
	2	0.9172	0.9243	0.7356	0.9206	0.9247	0.7356	0.9223	0.9219	0.7356
	5	0.9174	0.9237	0.7356	0.9204	0.9234	0.7356	0.9215	0.9214	0.7356
	10	0.9174	0.9238	0.7356	0.9204	0.9233	0.7356	0.9214	0.9214	0.7356
